# The Impact of Antifungal Stewardship on Clinical and Performance Measures: A Global Systematic Review

**DOI:** 10.3390/tropicalmed9010008

**Published:** 2023-12-29

**Authors:** Fares Albahar, Hamza Alhamad, Mohammad Abu Assab, Rana Abu-Farha, Lina Alawi, Sara Khaleel

**Affiliations:** 1 Department of Clinical Pharmacy, Faculty of Pharmacy, Zarqa University, P.O. Box 2000, Zarqa 13110, Jordan; halhamad@zu.edu.jo (H.A.); mabuassab@zu.edu.jo (M.A.A.); 2 Department of Clinical Pharmacy and Therapeutics, Faculty of Pharmacy, Applied Science Private University, P.O. Box 541350, Amman 11937, Jordan; r_abufarha@asu.edu.jo; 3 Department of Physiology and Pharmacology, Faculty of Medicine and Health Sciences, An Najah National University, Nablus P.O. Box 7, Palestine; lina.alawi@najah.edu; 4 Department of Pharmaceutical Sciences, Faculty of Pharmacy, Al Zaytoonah University, P.O. Box 130, Amman 11733, Jordan; sar9210027@ju.edu.jo

**Keywords:** antimicrobial stewardship, antifungal stewardship, systematic review, antimicrobial consumption, mortality, hospital length of stay, morbidity, cost-effectiveness

## Abstract

Background: Antimicrobial stewardship programs (ASP) have been proposed as an opportunity to optimize antifungal use. The antifungal resistance is a significant and emerging threat. The literature on antifungal stewardship (AFS) and its influence on performance and clinical outcome measures is scarce. This study aimed to examine global evidence of the impact of AFS on patients and performance measures. Methods: The “Preferred Reporting Items for Systematic Reviews and Meta-Analyses” (PRISMA) was used for the flow of identification, screening, eligibility, and inclusion. PubMed and MEDLINE were searched using the term ‘‘antifungal stewardship’’ on 15 February 2023. Search terms included antifungal stewardship, antimicrobial stewardship, candida, candidemia, candiduria, and invasive fungal disease. Of the 1366 records, 1304 were removed since they did not describe an antifungal stewardship intervention. Among the 62 full texts assessed, 21 articles were excluded since they were non-interventional studies and did not include the outcome of interest. Thus, 41 articles were eligible for systematic review. Eligible studies were those that described an AFS program and evaluated clinical or performance measures. Results: Of the 41 included studies, the primary performance measure collected was antifungal consumption (22 of 41), and mortality (22 of 41), followed by length of stay (11 of 41) and cost (9 of 41). Most studies were single-center, quasi-experimental, with varying interventions across studies. The principal finding from most of the studies in this systematic review is a reduction in mortality expressed in different units and the use of antifungal agents (13 studies out of 22 reporting mortality). Antifungal consumption was significantly blunted or reduced following stewardship initiation (10 of 22). Comparing studies was impossible due to a lack of standard units, making conducting a meta-analysis unfeasible, which would be a limitation of our study. Conclusion: It has been shown that AFS interventions may improve antifungal consumption and other performance measures. According to available published studies, antifungal consumption and mortality appear to be the possible performance measures to evaluate the impact of AFS.

## 1. Introduction

The effectiveness of current antibiotics is threatened by the quick global spread of resistant microorganisms [1,2]. Bacterial infections have reemerged as a hazard after a period of time in which patients with infections were treated with antibiotics [3,4]. Antibiotic abuse or overuse has been linked to the development of bacterial resistance [5]. Antimicrobial Resistance (AMR) results in increased mortality, morbidity, and prescribing costs. Therefore, the Society for Healthcare Epidemiology and the Infectious Disease Society of America published guidelines to optimize the use of antibiotics and contain AMR [6].

Antimicrobial stewardship (AMS) is defined as interventions developed to enhance and measure the appropriate use of antimicrobials by promoting the optimal usage of dosing regimen, dose, choice of antimicrobial, and duration [7]. The significance of AMS is that it has been globally recognized in improving patient outcomes (i.e., reducing mortality and morbidity), reducing antimicrobial consumption and costs, and reducing the development of antimicrobial resistance [8]. However, antifungal stewardship (AFS) received less global consideration compared to AMS despite its significance [8].

Although antimicrobial stewardship focuses on antibiotics, antifungal resistance is a growing and emerging threat [9]. For example, 70% of Candida glabatra and Candida auris species are resistant to fluconazole- and echinocandin [9,10]. Moreover, Candida auris was discovered in 2009 as an emerging multidrug-resistant pathogen, with cases or outbreaks reported in over 20 countries [10,11]. This is especially concerning given that Candida auris isolates are reportedly resistant to main classes of antifungal drugs [12]. Appropriate antifungal use is essential in fighting drug resistance [13]. AFS is the optimal selection of antifungal agents based on factors such as organism identity, patient toxicity profile and medication record, cost, and the potential of the emergence and spread of antifungal resistance [14].

Antifungal stewardship is a coordinated approach to monitoring and directing the appropriate use of antifungal agents to achieve optimal clinical outcomes and minimize selectivity and adverse events [14]. Antifungal guidelines are similar to those of antimicrobial stewardship programs (ASP), where the prescribing of antifungals is optimized by considering the spectrum of action, pharmacokinetics and pharmacodynamics (PK-PD), duration of use, and route of use [15]. Antifungals may already be used by existing anti-infective strategies (ASPs) due to their high cost, the potential for toxicity with long-term use, and the need for expertise to direct clinicians in prescribing [6]. Reducing healthcare costs is often a secondary effect of stewardship. As public awareness of the risks of antibiotic overuse increases, many anti-infectious strategies have initially focused on reducing antibiotic overuse [16,17,18]. However, the growing number of immunosuppressant patients at risk of opportunistic infections necessitates attention to other anti-infective classes [19].

Antimicrobial stewardship is about implementing coordinated interventions to enhance and evaluate the effective use of antimicrobials [20]. Invasive fungal infections are a significant cause of mortality and a global public health concern [21]. For example, in the United States, candidemia is only a small fraction of the burden caused by invasive candidiasis [22]. Also, hospitalization rates for invasive infections have increased, and the World Health Organization (WHO) has published a list of critical priority pathogens to support the global response against fungal infections; candida auris and fumigatus are both critical priority pathogens [23]. Public health efforts to address the threat of anti-fungal resistance are similar to those to combat antibiotic resistance.

Antimicrobial stewardship programs have well-documented evidence in optimizing the use of antimicrobials, thus improving patient outcomes, ensuring cost-effectiveness, reducing adverse outcomes such as reducing the incidence of C. difficile infections, and optimizing the use of healthcare resources [24,25]. Antimicrobial stewardship programs optimizes antifungals and minimizes the adverse and toxic effects of anti-fungal use and the possible emergence of resistant fungi [26]. Antifungal Stewardship (AFS) programs may improve performance measures and optimize antifungal consumption (i.e., potential economic savings) [27].

Antifungal consumption has been evaluated by the total anti-fungal prescriptions (TAP), which is defined by daily dose (DDD) and days of therapy (DOT) [28]. However, the long-term effects of AFS interventions are less well-understood and require further research, especially in settings such as critical care where multi-drug-resistant organisms (MDROs) are emerging [29,30]. Therefore, intensivists should balance the increased mortality associated with delaying therapy of microbiologically documented infections with the potential ecological damage caused by antimicrobial medications, including the selection and development of MDROs [29]. For example, a few hours’ delays in administering appropriate antimicrobial therapy in septic shock patients with sensitive causative pathogens would increase the mortality risk [31]. Also, this applies to other infections, such as those affecting the respiratory system (e.g., pneumonia, COVID-19), in which the use of an inappropriate initial antibiotic regimen would increase the risk of morbidity and mortality due to rising levels of bacterial resistance [14,15].

The recent COVID-19 pandemic and the risk of the patient becoming immunocompromised with a risk of systemic fungal infection highlighted the need for antifungal stewardship programs to prevent and fight unwarranted systemic infections [32,33]. Antifungal consumption during COVID-19 was evidenced to be increased [33,34]. However, a UK study reported that despite the COVID-19 pandemic’s effect on increasing antifungal consumption, the standards of care were good as a result of the presence of technology to facilitate antifungal stewardship programs [35]. However, tiny reductions in patient adherence were reported due to the switch from face-face to virtual meetings [35].

Establishing effective AFS aims to improve patient’s clinical outcomes, including mortality and morbidity, and performance measures, including antifungal consumption, cost, adverse drug reactions, and antifungal resistance. While AMS is extensively described in the literature [9,36,37,38,39], there is a scarcity of literature describing Antifungal Stewardship (AFS) as an emerging theme [9,39]. A systematic review of AFS interventions and performance measures in 2017 reported that antifungal consumption decreased by 11.8% to 71% and antifungal expenditure by 50% [39]. In 2017, a systematic review was conducted to examine the impact of AFS interventions in the United States and showed that AFS interventions could enhance patient outcomes and curtail antifungal use [9]. However, this study included 13 studies from the United States only [9]. However, this study included 13 studies from the United States only [9]. Therefore, updated and recent evidence about the impact of AFS interventions is necessary from studies reported in other countries globally. This systematic review aimed at examining and summarizing studies reporting the evidence of the global impact of AFS and available interventions on clinical and performance measures. This would help inform and support healthcare professionals with the latest evidence, improve patient outcomes and safety, and reduce healthcare financial expenditures.

## 2. Materials and Methods

### 2.1. Search Strategy

**Figure 1:**
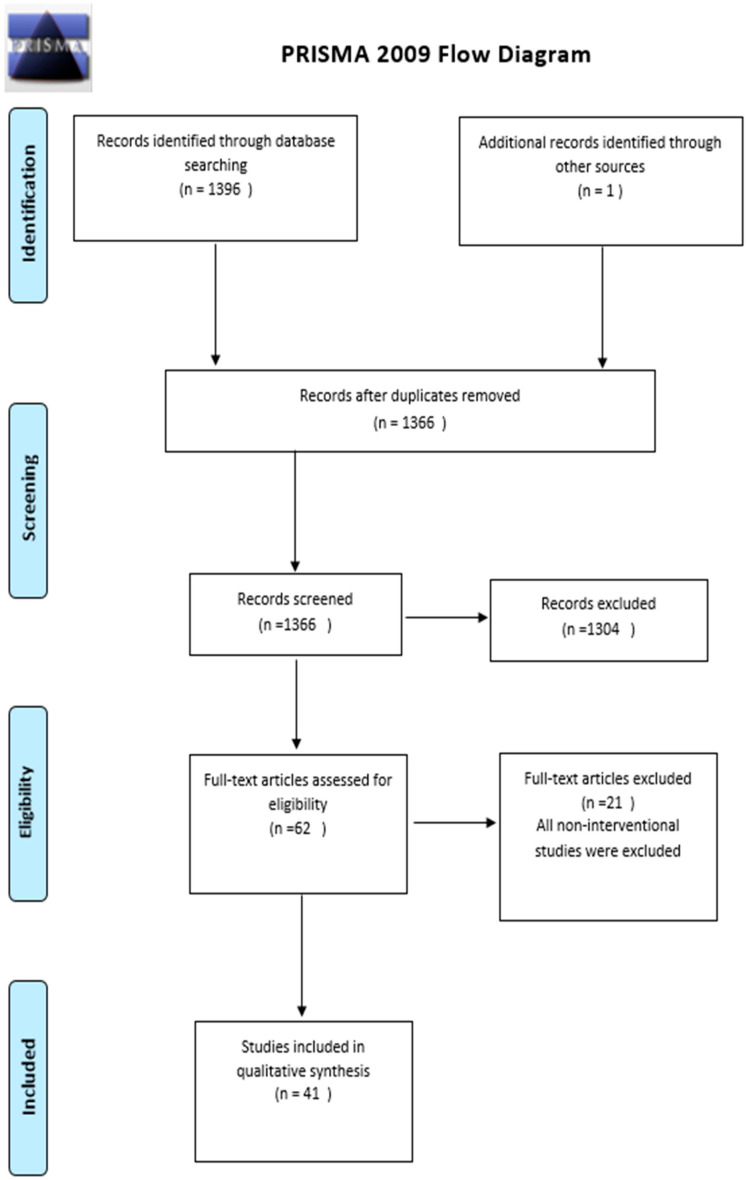
Literature search scope using the PRISMA flow chart adapted from the PRISMA Group [40].

The literature search was conducted using EMBASE and PubMed online databases to pursue articles related to antimicrobial and antifungal stewardship. Moreover, the reference lists of relevant articles related to the impact of antifungal stewardship on clinical and performance measures were searched to increase completeness. The last search was performed on 17 February 2023. Medical Subject Heading (MeSH) terms were initially identified using the PubMed-linked MeSH database. The selected MeSH terms were “antifungal stewardship”, “antimicrobial stewardship”, “candida, invasive fungal”, “candidemia”, “candiduria”, and “aspergillosis”. Three reviewers (HA, FA, RA) assessed the titles and abstracts of retrieved references to establish potential inclusion eligibility. The full texts of potential studies were reviewed to see if they met the review inclusion criteria. Bibliographies of retrieved papers and prior systematic reviews were checked to find other articles that this search approach may have overlooked. A total of 1366 records were identified; one record was obtained using the snowballing approach. Of the 1366 records, 1304 were removed since they did not describe an antifungal stewardship intervention. Among the 62 full texts assessed, 21 articles were excluded since they were non-interventional studies and they did not include the outcome of interest. Thus, 41 articles were eligible for systematic review (Figure 1).

### 2.2. Eligibility Criteria

The eligibility criteria were set during the search process for the related articles. Studies that described an AFS included an intervention, clinical performance, and outcome measures such as mortality and morbidity (i.e., hospital length of stay, antimicrobial consumption, cost, antifungal therapy use, and effectiveness). Exclusions were made for non-English studies, reviews, and studies that did not include intervention, performance, or clinical outcome measures. A wide range of outcomes was measured, including appropriate fungal choice, time to therapy, cost, antifungal consumption, mortality, and length of stay.

### 2.3. Study Selection

Study selection was completed by two researchers (FA and HA) using Preferred Reporting Items for Systematic Reviews and Meta-Analyses (PRISMA) (Appendix A) flow of identification, screening, eligibility, and inclusion. Abstracts were uploaded to MEDLINE to determine whether publications were eligible after the records were checked for duplicates. If the abstract did not provide sufficient information to determine eligibility, full texts were downloaded from the university library. Using a snowballing strategy, relevant reviews and references of eligible publications were searched to make the search more thorough. Two researchers (FA and HA) separately evaluated full-text papers to settle any differences regarding inclusion, and following discussion, a consensus was reached.

### 2.4. Data Extraction

A custom data extraction form was developed to meet the review’s special requirements. One reviewer (FA) extracted and confirmed data on the study design, participants, interventions, comparators, outcomes, and key findings (HA). Disagreements were settled through consensus, with the assistance of a third investigator (RA). Two reviewers screened all titles and abstracts identified in the literature search. Abstracts were eligible if they fulfilled the inclusion criteria, and full-text articles were additionally reviewed and discussed by the two researchers where a data collection form was used to collect information from the retrieved studies, including; the study title, year of publication, author, objectives, design, patient population, duration, site, intervention description, and findings on outcomes of interest were used to extract the data. In addition, another researcher reviewed the extracted data to verify the necessity. Any conflict on data inclusion was confirmed through discussion between all of the researchers.

### 2.5. Synthesis of Results

The Preferred Reporting Items for Systematic Reviews and Meta-Analyses (PRISMA) checklist [40] was used to guide the systematic review (Appendix A). The extracted data were summarized descriptively based on intervention variability, patient populations, and outcome measures. A narrative process was used to describe data extracted from full-text articles. The initial search of the databases resulted in 1396 articles in total, with one article identified through other sources. After removing the duplicates, 1366 were screened, and 1304 articles were removed. The 62 full-text articles were assessed, in which 41 articles were included in the qualitative synthesis.

### 2.6. Quality Assessment of Included Studies

Case-control, cohort, randomized controlled trials, and case series studies were critically evaluated using the National Institutes of Health (NIH) quality evaluation method [41]. Using the appropriate technique based on the study design, two reviewers independently evaluated the quality of each study. Studies were rated on a scale of good, fair, or poor, with a score of two being considered good (11–14 out of 14 questions), a score of one considered acceptable (5–10 out of 14 questions), and a score of zero being considered poor (0–4 out of 14 questions). Additionally, each included study’s quality was evaluated separately by two researchers. If their assessments of the studies were different, both authors discussed the article to come to a decision.

## 3. Results

The search yielded 1366 candidate studies. Of the 1366 records, 1304 were removed since they did not describe an antifungal stewardship intervention. Among the 62 full texts assessed, 21 articles were excluded since they were non-interventional studies and did not include the outcome of interest. Thus, a total of 41 articles comprising data from different countries from around the world (except for 4 studies which did not report the country); USA [2,42,43,44,45,46,47,48,49,50,51,52,53,54,55], UK [56,57,58,59], Ireland [60], Germany [61,62], Spain [63,64,65,66,67], France [68,69,70,71,72], Italy [73], Thailand [74], Japan [75,76] were included and reviewed. More than half of the studies were published in 2014 or later. The first study describing an antifungal stewardship intervention was published in 2004 [68], and its main objective was to evaluate the systematic mycological screening performed on all patients admitted to the Surgical ICU [68]. A summary of the study’s characteristics, methodologies, clinical performance, and outcome measures that are included in this systematic review are presented in Appendix B.

### 3.1. Study Characteristics

Of the included studies, 22 studies reported clinical outcomes such as mortality. These studies are summarized in Appendix B. The remaining studies reported different outcomes, such as cost, appropriateness of antifungal use, and consumption. All studies were single-centered and quasi-experimental in design, with the earliest publication in 2004 [68]. Data that were not related to antifungals were not included in the review.

### 3.2. Interventions

The stewardship interventions differed across the studies, but common stewardship interventions included audit, feedback, and preauthorization requirements [43,53,54]. Interventions ranged from applying a stewardship care bundle, guideline development, audit and feedback, and preauthorization. For instance, six studies were based on introducing diagnostic tools for detecting candida species [49,51,56,60,77,78]. Intervention types and implementation are presented in (Appendix A).

### 3.3. Outcome Measures

#### 3.3.1. Mortality

Twenty-two studies reported clinical outcomes such as mortality [2,42,47,48,49,50,51,56,57,60,63,64,65,68,69,70,73,75,76,77,78,79]. Thirteen studies were associated with lower mortality rates in the intervention group. In one study, there was a significant difference in mortality between the intervention and non-intervention groups, where the 90-day mortality was 29% [222/776] in the intervention group compared to 51% [468/915] in the non-intervention group, *p* < 0.0001 [50]. In this retrospective, single-center cohort analysis, the medical records of all patients with a candida bloodstream infection were examined to compare 90-day all-cause mortality between people who had and did not have an infectious disease consultation [50].

#### 3.3.2. Hospital Length of Stay

Eleven studies reported hospital length of stay [2,47,48,49,51,52,53,60,63,75,80]. None of these studies showed a clinically significant reduction in hospital length of stay. In one study, the hospital length of stay was ten days in the intervention group compared to eleven days in the non-intervention group, but it was not statistically significant (*p* = 0.68) [2]. This quasi-experimental study was conducted to evaluate how an ASP pharmacist’s interventions affected the length of time it took patients with candidemia to receive effective antifungal treatment. Comparing patients from 2008 (*n* = 85 pre-intervention) and 2010 (*n* = 88 post-intervention), the time to effective therapy was much faster in the post-intervention group (median 13.5 versus 1.3 h, *p* = 0.04) and was given to more patients (67 (88%) vs. 80 (99%), *p* = 0.008) [2].

#### 3.3.3. Antimicrobial Consumption

Twenty-two studies reported on antifungal consumption [43,44,47,50,51,52,53,54,55,58,61,64,65,66,67,68,69,71,74,79,80], of which ten studies showed a decrease in the consumption of antifungals used [50,54,55,61,64,65,66,68,71,74]. One study evaluated the effect of an antifungal stewardship program on the use of all antifungals (except for fluconazole, on candidemia mortality) and reported an increase in the use of antifungals [73]. Researchers looked back at the medical records of patients with candidemia documented between 2012 and 2014 to assess the effects of several factors on 30-day in-hospital mortality [73]. Data on 276 individuals with verified candidemia were examined; 200 (72%) received no treatment, whereas 76 (28%) received infectious diseases consultation [73]. Fifty-two individuals (26%) in the group without infectious diseases consultation received no antifungal medication [73]. With or without infectious disease consultation, the 30-day in-hospital mortality was 37% compared to 20% (*p* = 0.011) [73]. Various units were used to describe antifungal consumption, including defined daily doses per 1000 patient days or 100 admissions, days of therapy per 1000 patient days, median days of therapy, and doses per 1000 patient days [43,53,69]. The quantitative comparison between the studies was impossible due to the lack of common units.

#### 3.3.4. Cost

Eighteen studies reported on the antifungal cost [2,43,45,48,49,51,53,57,58,59,61,64,66,69,73,74,75,80], of which 12 studies showed a reduction in the cost of using antifungal agents [2,43,45,49,51,53,55,59,61,64,66,74]. One study reported an increment in the cost of using antifungals [73]. Various units were used to describe the cost of antifungals, making direct quantitative comparison difficult between the studies.

#### 3.3.5. Antifungal Therapy Use and Effectiveness

Fifteen studies reported on antifungal therapy use and effectiveness [2,44,46,54,56,62,63,65,69,72,74,75,76,77,81]. Antifungal therapy use was described in terms of adherence to treatment guidelines [54,72], the appropriateness of the antifungal treatment [2,56,62,65,76,81], and the antifungal consumption [75]. The antifungal therapy effectiveness was described as; fewer days of therapy [46,63,74,77], no change in therapy is recommended [44], and cost-effectiveness [69].

## 4. Discussion

The literature is rich with studies evaluating the impact of AMS on patient and performance measures. However, there is a paucity of literature evaluating (AFS) [14,30,46,57,64,65,66,71,72,74,75,77]. This warrants conducting a systematic review to update the policymakers and healthcare professionals about the current status of the clinical and performance measures related to antifungal use, effectiveness, cost-effectiveness, and appropriateness. This systematic review addresses that gap. The principal finding from most of the studies in this systematic review is a reduction in mortality expressed in different units and the use of antifungal agents. Also, other studies reported on the cost-effectiveness and appropriateness of antifungal therapy.

Antifungal stewardship programs are an integral part of the antimicrobial stewardship program, given the rise in antifungal resistance and poor clinical outcomes [9]. The multidrug-resistant Candida curis is one of the challenges impacting patients’ clinical outcomes [51]. Therefore, additional AFS interventions and programs are needed to contain antifungal resistance properly. It has been shown that AFS interventions were implemented in tertiary care and teaching hospitals [9,57]. This would explain the frequent use of broad-spectrum antifungals for critically ill patients admitted in such healthcare settings and the availability of facilities and resources needed to implement AFS. The multidisciplinary team’s role in containing invasive fungal infections is debatable [55]. It should contain an infectious disease physician, a clinical pharmacist, and a clinical microbiologist. However, only 5 of the 41 studies in this systematic review reported a complete antifungal stewardship team [2,64,65,66,74]. A hospital epidemiologist, an infection control professional, and an information system specialist are also included in the antimicrobial stewardship team, according to IDSA guidelines [53].

Notably, none of the included studies contain an antimicrobial stewardship team with such healthcare professionals, and the recommendations of these studies do not endorse including these staff. Moreover, pharmacists played an integral role in the antimicrobial stewardship team, and their absence from the team was associated with a higher rate of inappropriate antimicrobial prescribing and a longer duration of treatment [43]. The stewardship interventions differed across studies, but common stewardship interventions included audit, feedback, and preauthorization requirements [43,53,54]. Six studies were based on introducing diagnostic tools for detecting candida species [49,51,56,60,77,78]. Mortality and antifungal consumption were the most commonly reported outcomes in this systematic review. The majority of studies showed a reduction in mortality expressed in different units. Various approaches were used to express consumption, including defined daily doses and days of therapy. The use of antifungal days of therapy is the most selected metric, according to IDSA, as it can be used for pediatrics and is not affected by dose adjustments [9].

Interestingly, all studies showed reduced use of antifungal agents. Such reduction in antifungal use was apparent in studies reporting both overall antifungal utilization and those focusing on specific antifungal classes or drugs. Although AFS can positively impact antifungal consumption, the prescribing quality within these studies is unclear. Only four studies reported on the appropriateness of antifungal use. The majority of studies did not evaluate the suitability of antifungal prescribing as a process outcome.

Previous research showed a high proportion of inappropriate antifungal agent use, including inadequate dosages or indications [82,83]. Given the overtreatment with antifungal therapy and the rise in resistance, there should be a greater focus on compliance with guideline recommendations as a reported performance measure.

Establishing the impact of AFS interventions on clinical outcomes such as mortality should be a primary focus, along with reporting antifungal utilization and other process outcomes. Half of the included studies in this systematic review evaluated clinical outcomes, including in-hospital or 30-day mortality and overall hospital length of stay. ASPs were associated with a considerable reduction in hospital length of stay. However, these findings were based on only six studies [2,49,51,52,53,60]. Two more studies did not show any change in the length of stay [47,48]. The scarcity of studies (i.e., 8 out 41) that evaluate the impact of ASPs on hospital length of stay would necessitate further studies to be conducted to strengthen the evidence.

Findings from this systematic review support previous reviews in which stewardship programs do not negatively influence patient care levels by focusing antifungal therapy on patients who need it. However, similar to antimicrobial stewardship, AFS programs must evaluate clinical outcomes and show care improvements to justify additional resources beyond the cost savings associated with decreased antifungal consumption. Despite the significance of antifungal stewardships for patients, policymakers, and healthcare professionals, the first study in this systematic review describing an antifungal stewardship intervention was published in 2004. Also, more than half of the studies were published in 2014 or later. This would provide a clear picture of the need to conduct more research related to antifungal stewardship that would be used by stakeholders (policymakers, healthcare professionals, and patients) to influence the effective use of antifungals. The significance of this systematic review is that it includes updated and recent evidence from around the globe exploring healthcare systems worldwide compared to the previous two systematic reviews of AFS [9,39]. However, our study has many limitations. The major limitation is the scarcity of literature and evidence to support AFS programs. Studies focusing on AFS programs were primarily published after 2010, consistent with this concept’s emergence [74]. Another significant limitation is that most included studies were non-randomized, primarily single-center, quasi-experimental designs.

Furthermore, specific recommendations were drawn from studies with small numbers of patients. Moreover, the heterogeneity of the included studies makes conducting a meta-analysis very difficult as the outcomes measured are reported in different units. These limitations warrant focusing on and conducting more antifungal stewardship-related research to gain more evidenced-based insights about the rational use of antifungals, thus helping policymakers develop and update the antifungals protocols and guidelines and allowing infectious consultants and other healthcare professionals to provide rational antifungal treatment. Therefore, raising awareness about the significance of antifungal stewardship is paramount with stakeholders (i.e., healthcare providers, prescribers, policymakers, and patients) education, and developing and implementing national and international antifungal guidelines would be the starting point.

## 5. Conclusions

Findings from this systematic review shed light on the impact of antifungal stewardship on clinical and performance measures. Mortality was reported to be reduced in about half of the studies that reported mortality, along with reduced use of antifungal agents. This would signify the importance of effective antifungal utilisation (i.e., consumption metrics) based on appropriate use and adherence to antifungal guidelines on reducing mortality rate and improving morbidity-related clinical measures. Also, none of the included studies contain an antimicrobial stewardship team, and the recommendations of these studies do not endorse including these staff. This is significant, in which a multidisciplinary team of AFS is paramount for the success of AFS. All AFS interventions included in this systematic review impacted clinical and performance measures, including consumption and cost. Future works are paramount, considering the scarce antifungal stewardship-related literature. They should focus on conducting high levels of evidence-based medicine such as systematic reviews, meta-analysis, and randomized controlled trials to evaluate AFS on clinical and performance measures and developing guidelines for AFS implementation, as is the case for AMS. Also, research should focus on new antifungals and their role in devising empirical treatment, which will impact the future of antifungal stewardship.

## Data Availability

The data presented in this study are available on request from the corresponding author.

## Appendix A

**Table A1:** PRISMA 2020 Checklist.

Section and Topic	Item #	Checklist Item	Location Where Item Is Reported
**TITLE**			
Title	1	Identify the report as a systematic review.	Page 1
**ABSTRACT**			
Abstract	2	See the PRISMA 2020 for Abstracts checklist.	Pages 1 & 2
**INTRODUCTION**			
Rationale	3	Describe the rationale for the review in the context of existing knowledge.	Page 3
Objectives	4	Provide an explicit statement of the objective(s) or question(s) the review addresses.	Page 3
**METHODS**			
Eligibility criteria	5	Specify the inclusion and exclusion criteria for the review and how studies were grouped for the syntheses.	Page 3
Information sources	6	Specify all databases, registers, websites, organisations, reference lists and other sources searched or consulted to identify studies. Specify the date when each source was last searched or consulted.	Page 3
Search strategy	7	Present the full search strategies for all databases, registers and websites, including any filters and limits used.	Page 3
Selection process	8	Specify the methods used to decide whether a study met the inclusion criteria of the review, including how many reviewers screened each record and each report retrieved, whether they worked independently, and if applicable, details of automation tools used in the process.	Page 4
Data collection process	9	Specify the methods used to collect data from reports, including how many reviewers collected data from each report, whether they worked independently, any processes for obtaining or confirming data from study investigators, and if applicable, details of automation tools used in the process.	Page 4
Data items	10a	List and define all outcomes for which data were sought. Specify whether all results that were compatible with each outcome domain in each study were sought (e.g., for all measures, time points, analyses), and if not, the methods used to decide which results to collect.	Page 4
	10b	List and define all other variables for which data were sought (e.g., participant and intervention characteristics, funding sources). Describe any assumptions made about any missing or unclear information.	Page 4
Study risk of bias assessment	11	Specify the methods used to assess risk of bias in the included studies, including details of the tool(s) used, how many reviewers assessed each study and whether they worked independently, and if applicable, details of automation tools used in the process.	Pages 4–5
Effect measures	12	Specify for each outcome the effect measure(s) (e.g., risk ratio, mean difference) used in the synthesis or presentation of results.	N/A
Synthesis methods	13a	Describe the processes used to decide which studies were eligible for each synthesis (e.g., tabulating the study intervention characteristics and comparing against the planned groups for each synthesis (item #5)).	Page 3
	13b	Describe any methods required to prepare the data for presentation or synthesis, such as handling of missing summary statistics, or data conversions.	Page 4
	13c	Describe any methods used to tabulate or visually display results of individual studies and syntheses.	Page 4
	13d	Describe any methods used to synthesize results and provide a rationale for the choice(s). If meta-analysis was performed, describe the model(s), method(s) to identify the presence and extent of statistical heterogeneity, and software package(s) used.	Page 4
	13e	Describe any methods used to explore possible causes of heterogeneity among study results (e.g., subgroup analysis, meta-regression).	N/A
	13f	Describe any sensitivity analyses conducted to assess robustness of the synthesized results.	N/A
Reporting bias assessment	14	Describe any methods used to assess risk of bias due to missing results in a synthesis (arising from reporting biases).	Pages 4–5
Certainty assessment	15	Describe any methods used to assess certainty (or confidence) in the body of evidence for an outcome.	N/
**RESULTS**			-
Study selection	16a	Describe the results of the search and selection process, from the number of records identified in the search to the number of studies included in the review, ideally using a flow diagram.	Page 5
	16b	Cite studies that might appear to meet the inclusion criteria, but which were excluded, and explain why they were excluded.	Pages 5 & 8
Study characteristics	17	Cite each included study and present its characteristics.	Pages 9–23
Risk of bias in studies	18	Present assessments of risk of bias for each included study.	Page 5
Results of individual studies	19	For all outcomes, present, for each study: (a) summary statistics for each group (where appropriate) and (b) an effect estimate and its precision (e.g., confidence/credible interval), ideally using structured tables or plots.	Pages 9–23
Results of syntheses	20a	For each synthesis, briefly summarise the characteristics and risk of bias among contributing studies.	Pages 9–23
	20b	Present results of all statistical syntheses conducted. If meta-analysis was carried out, present for each the summary estimate and its precision (e.g., confidence/credible interval) and measures of statistical heterogeneity. If comparing groups, describe the direction of the effect.	N/A
	20c	Present results of all investigations of possible causes of heterogeneity among study results.	N/A
	20d	Present results of all sensitivity analyses conducted to assess the robustness of the synthesized results.	N/A
Reporting biases	21	Present assessments of risk of bias due to missing results (arising from reporting biases) for each synthesis assessed.	N/A
Certainty of evidence	22	Present assessments of certainty (or confidence) in the body of evidence for each outcome assessed.	Pages 6 & 7
**DISCUSSION**			
Discussion	23a	Provide a general interpretation of the results in the context of other evidence.	Page 25
	23b	Discuss any limitations of the evidence included in the review.	Page 26
	23c	Discuss any limitations of the review processes used.	Page 26
	23d	Discuss implications of the results for practice, policy, and future research.	Pages 26–27
**OTHER INFORMATION**			
Registration and protocol	24a	Provide registration information for the review, including register name and registration number, or state that the review was not registered.	Not registered
	24b	Indicate where the review protocol can be accessed, or state that a protocol was not prepared.	Not prepared
	24c	Describe and explain any amendments to information provided at registration or in the protocol.	N/A
Support	25	Describe sources of financial or non-financial support for the review, and the role of the funders or sponsors in the review.	Page 26
Competing interests	26	Declare any competing interests of review authors.	Page 26
Availability of data, code and other materials	27	Report which of the following are publicly available and where they can be found: template data collection forms; data extracted from included studies; data used for all analyses; analytic code; any other materials used in the review.	Pages 9–23

## Appendix B

**Table A2:** A summary of the characteristics of the included studies.

Author, Year	Context	Interventions	Duration of Intervention	Outcomes (Pre-Interventions vs. Post-Interventions)				
				Mortality	Morbidity			
					Length of Stay	Costs	Antifungal Consumption	Effective Antifungal Therapy
Reed, Erica E., et al. (2014) [2]	Academic center located in Columbus, Ohio, USA.	Candedimeia guidelines	1 January 2008 to 31 December 2010.	There was no significant difference in the in-hospital mortality [16 (19%) vs. 26 (30%) patients, *p* = 0.11]	infection-related LOS [10 (7–15.5) vs. 11 (7–17) days, *p* = 0.68]	hospital costs during candidemia [$25,697 (15,645–42,870) vs. $31,457 ($16,399–83,649), *p* = 0.25]	Not reported	Effective antifungal therapy 67% vs. 80%, *p* = 0.008
Swoboda et al. (2009) [61]	University hospital Heidelberg, Germany	To outline the impact of standardized form on antifungal stewardship	2005–2007. 18 months before and 18 months after implementation of guidelines	Not reported	Not reported	Data analysis revealed a decrease in costs by 50%.	Intervention was correlated with a significant reduction in use of antifungal agents.	Not reported
López-Medrano et al. (2013) [64]	University-affiliated Hospital 12 de Octubre, Madrid, Spain	To review prescriptions and make non-compulsory recommendations Request handling = all antifungal prescriptions checked every working day from the computerized system of the pharmacy	2008–2009. 24 months	The programme was not related to significant increases in the incidence of 12-month mortality in patients with filamentous fungal infections	Not reported	Expenditure on antifungals was reduced by US $370,681.78 (11.8% reduction)	The DDDs of intravenous voriconazole and caspofungin were reduced by 31.4% and 20.2%, respectively	Not reported
Standiford et al. (2012) [45]	Tertiary care academic medical centre, Baltimore, USA	To evaluate the cost before, during and after the antifungal stewardshipprogramme	2001–2010. 2001 before implementation/2002–2008 during implementation/2009–2010 after implementation	Not reported	Not reported	↓ 45.8%	Not reported	Not reported
Antworth et al. (2013) [46]	Academic Hospital, 930 beds, Michigan, USA	To analyse the impact of a care bundle by antimicrobial stewardship team on the management of candidaemia	2010–2011. 7 months non-interventional/7 months interventional	Not reported	Not reported	Not reported	Not reported	Fewer excess days of therapy (5 vs. 83 days
Mondain et al. (2013) [72]	Teaching tertiary care hospital, 1800 beds, Nice, France	To describe and assess antifungal stewardship impact on antifungal prescriptions	2005–2010. 5 years	Not reported	Not reported	Not reported	Not reported	88% adherence to treatment guidelines
Alfandari et al. (2014) [71]	University Hospital, Lille, France	To implement antifungal stewardship	2009–2010. 24 months	Not reported	Not reported	Not reported	↓ 40% of antifungal consumption	Not reported
Valerio et al. (2015) [66]	Tertiary Hospital, Gregorio Maranon, Madrid, Spain	To describe a bedside non-restrictive antifungal stewardship and evaluate its economic impact	October 2010–September 2012. 12 months non-interventional/1 year and 2 months interventional	Not reported	Not reported	↓ 21.7%	↓ DDD 17%	Not reported
Cook and Gooch et al. (2015) [54]	Tertiary care teaching Hospital, 904 beds, Greenville, USA	To assess the antimicrobial stewardship impact on antimicrobial use, including antifungals	2001–2013. 13 years	Not reported	Not reported	Not reported	↓ 71%	Increase of 9.2% of adherence to guidelines
Ramos, Antonio, et al. (2015) [65]	the Hospital Puerta de Hierro, Spain	Programme review of restricted antifungals	Between 1 October 2012 and 31 May 2013	Mortality 17% vs. 30% (*p* = 0.393)	Not reported	Not reported	DDDs per occupied beddays decreased from 5.06 to 2.92	inappropriate treatment 70 vs. 28%
Piarroux, Renaud, et al. (2004) [68]	Surgical ICUin a university affiliated Hospital, France	Systematic mycological screening was performed on all patients admitted to the SICU	From August 1998 to November 2002	76 [16.7] vs. 73 [15.3], *p* = 0.55	Not reported	Not reported	9.4 ± 9.1 vs. 8.8 ± 7.3 *p* = 0.25	Not reported
Apisarnt hanarak, (2010) [74]	Thammasat university hospital, Thailand	Antifungal drug use for treatment of candidiasis among inpatients	3 years	Not reported	Not reported	Total cost savings were US $31,615 during the 18-month post-intervention period	59% reduction in antifungal prescriptions (from 194 to 80 prescriptions per 1000 hospitalizations; *p* < 0.001	Antifungal use decreased (from 71% to 24%; *p* < 0.001), patient-days; *p* < 0.001).
Huang, Angela M., et al. (2013) [49]	University of Michigan Health System and College of Pharmacy, USA	The Matrix Assisted Laser Desorption Time of Flight Combined With Antimicrobial Stewardship Team	3-month period (1 September–30 November 2012)	Mortality (20.3% vs. 14.5%)	The ICU length of stay was (14.9 vs. 8.3 days)	The total hospital costs ($45,709 vs. $26,126, *p* = 0.009)	Not reported	
Mejia-Chew, Carlos, et al. (2019) [50]	Barnes Jewish Hospital (St Louis, MO, USA)	Infectious disease consultation	between 1 January 2002, and 31 December 2015	90-day mortality (29% [222/776] vs. 51% [468/915]; *p* < 0.0001).	Not reported	Not reported	Total duration of antifungal therapy (18 [IQR 14–35] vs. 14 [6–20] days; *p* < 0.0001)	Not reported
Murakami, Minoru, et al. (2018) [76]	Saku Central Hospital, located in Nagano Prefecture, Japan.	Significantly improved adherence to the guidelines for management of candidemia	Between November 2006 and October 2012.	Mortality at 30 day 7 (23.3%) vs. 11 (23.9%), *p* = 0.91	Not reported	Not reported	Not reported	Appropriate empirical antifungal therapy (100% vs. 60.0%; proportion ratio 1.67 [95% CI 1.24–2.23]),
Heil, Emily L., et al. (2012) [51]	A tertiraly care centre, USA	A rapid peptide nucleic acid fluorescence in situ hybridization (PNA FISH) assay with an antimicrobial stewardship interventions	(26 June 2009–19 September 2010)	Mortality, no. (%) pts 19 (31%) vs. 5 (24%), *p* > 0.99	Median (IQR) length of hospital stay, days 25 (16 to 33) vs. 12 (9 to 30), *p* = 0.82	Savings of approximate $415 per patient.	Total treatment duration, days 14 (13 to 18) vs. 17 (14 to 19)	Not reported
Guarascio, Anthony J., et al. (2013) [55]	West Virginia University Healthcare, USA	Antifungal bundle in the intensive care unit	Six-month time period from February 2011 to July 2011	Not reported	Not reported	Cost savings of approximately $1013 per patient.	A significant reduction in median days of caspofungin therapy (4.00 vs. 2.00 days, *p* = 0.001) was found in the bundle group. Most of this reduction in use was realized in the medical ICU (*p* = 0.002) as opposed to the surgical ICU (*p* = 0.188)	Not reported
Storey, Donald F., et al. (2012) [53]	Community hospital located in metropolitan, USA	Automatic vancomycin dose optimization and a pneumonia order set. severe sepsis order sets, and a parenteral to oral conversion protocol	16-month intervention period (September 2009–December 2010)	Not reported	ALOTS, mean(SD), days 3.9 (0.3) vs. 3.6 (0.3) *p* = 0.118	Antimicrobial acquisition cost per admission 87.0 vs. 59.4 (*p* = 0.013)	There was a 22% decrease in defined daily doses per 100 admissions (*p* = 0.006)	Not reported
Jenkins, Timothy C., et al. (2015) [43]	Denver Health Hospital, USA	The ASP used in a hospital with low baseline antibiotic use	6.25-year period (1 July 2008–30 September 2014)	Not reported	Not reported	The antibiotic expenditures dcreased significantly during the ASP (−$295.42/1000 PD per quarter, *p* = 0.002).	The total antibacterial and antipseudomonal use were decreasing (−9.2 and −5.5 DOT/1000 PD per quarter, respectively).	Not reported
Menichetti, Francesco, et al. (2018) [73]	Pisa tertiary- care, University hospital, Italy	The infectious diseases consultation as a part of an antifungal stewardship programme on candidemia outcome	January 2012–December 2014	The 30-day in-hospital mortality was 37% for patients cared for without Infectious disease consultation (IDC) and 20% for those treated by IDC, a statistically significant difference (*p* = 0.011)	Not reported	Overall, the antifungal cost rose from £387,000 in the 2012 to £595,000 in 2014, an increase of £207,000 (53%)	An increase in use of fluconazole (from 3.1 to 4.3 DDD/100 bed days) and echinocandins (from 0.22 to 0.35) while voriconazole use decreased (from 0.25 to 0.18).	Not reported
Siegfried, Justin, et al. (2017) [44]	NYU Langone Medical Center, USA	ASP coverage of nighttime, holiday, and weekend shifts is often provided by infectious diseases (ID) medical fellows	Not reported	Not reported	Not reported	Not reported	Decrease in aggregate antimicrobial use from 799.3 ± 46.8 to 740.7 ± 17.3	No change in therapy recommended 51 (59%) vs. 50 (66%)
Micallef, C., et al. (2015) [59]	Cambridge University Hospitals NHS Foundation in the East of England	The careful selection of antimicrobials based on patient profile, target organism, toxicity, costs	12 month study	Not reported	Not reported	cost saving of £180,000.	Not reported	Not reported
Benoist et al. (2019) [70]	French University Hospital, France	Comparing the clinical outcomes of patients with candidaemia before and after the implementation of an antifungal stewardship program (AFSP).	4 years: 2 years before and 2 years after	The 3 months mortality rate decreased from 36.4% in the first period into 27.0% in the second period (*p* = 0.4).	Not reported	Not reported	Not reported	Not reported
Ito-Takeichi et al. (2019) [77]	Not reported	To assess the impact of implementing an antifungal stewardship with monitoring of β D-Glucan values on antifungal use and clinical outcomes	2013–2019, 6 years	The rate of 60-day mortality associated with Candida bloodstream infection tended to be reduced, from 42.9% (15/35) to 18.2% (4/22) (*p* = 0.081) compared to pre intervention group.	Not reported	Not reported	Not reported	Parental antifungal use was reduced significantly (*p* = 0.006). Clinical failure reduction, from 80.0% (28/35) to 36.4% (8/22) (*p* < 0.001)
Lachenmayr et al. (2019) [62]	German tertiary care hospital, Germany	AFS measures included medical training (two sessions), a pocket card summarising main recommendations for antifungal use, and daily pharmaceutical counselling on the ward.	6 months	Not reported	Not reported	Not reported	Not reported	significant increase in dosage accuracy (+19.3%; *p* < 0.05) and correct choice of drug (+15.9%; *p* < 0.05) was noted,
Rautemaa-Richardson et al. (2018) [56]	Referral tertiary teaching hospital, UK	Invasive fungal infection guidelines utilizing an informative biomarker [serum β-1-3-d-glucan (BDG)] were implemented	4 month audit in 2014 and 2016	Mortality due to invasive candidosis was reduced by 58%	Not reported	Not reported	Not reported	The number of inappropriate initiations of antifungals reduced by 90%.
Nwankwo et al. (2018) [58]	Tertiary cardiopulmonary hospital in England, UK	Antifungal stewardship programme targeting antifungals	Not reported	Not reported	Not reported	Reduction in monthly antifungal expenditure (*p* = 0.005) by £130,000 per month	Significant reduction in antifungal use, measured as the defined daily dose/100 bed days (*p* = 0.017)	Not reported
Rac et al. (2018) [42]	Urban, tertiary academic medical center USA	A one-time targeted candidemia intervention on time to initiation of adequate therapy compared to standard of care	Preintervention (1 August 2012 to 31 July 2014) and post-intervention (1 October 2014 to 30 September 2016	No change in-hospital mortality (*p* = 0.761)	Not reported	Not reported	Not reported	Not reported
Whitney et al. (2019) [57]	London Teaching Hospital, UK	Audit of antifungals by infectious diseases consultant and clinical pharmacist	2010–2016	Inpatient mortality was not affected	Not reported	expenditure initially reduced by 30% then increased to 20%	Not reported	Not reported
Martín-Gutiérrez et al. (2020) [79]	Not reported	Comprehensive antimicrobial stewardship program on antifungal use	9-year period	Mortality reduced from 0.044–0.017	Not reported	Not reported	Reduction of antifungal consumption by 38%	Not reported
Hebart et al. (2009) [78]	Not reported	Empirical plus PCR-based vs. empirical liposomal amphotericin B treatment	Not reported	Survival curves showed better survival until day 30 (mortality 1.5 vs. 6.3%; *p* = 0.015), but there was no difference at day 100	Not reported	Not reported	Not reported	Not reported
Cordonnier et al. (2009) [69]	13 French hospitals, France	Multicenter, open-label, randomized noninferiority trial, empirical antifungal therapy vs. pre-emptive one	3 years	Survival was not lower with preemptive treatment (95.1%) than with empirical treatment (97.3%), and the 95% CI for the difference was −5.9% to 1.4%.	Not reported	The total number of days of antifungal treatment and were significantly lower	Antifungal therapy was given for isolated persistent or recurrent fever to 55 (59.8%) of 92 patients in the empirical treatment arm and 1 (1.8%) of 56 patients in the preemptive treatment arm (*p* < 0.001)	The mean costs of antifungal drugs were significantly lower for the preemptive treatment group.
Morrissey et al. (2013) [81]	Not reported	Galactomannan and PCR versus culture and histology for directing use of antifungal treatment	26 weeks	Not reported	Not reported	Not reported	Not reported	39 patients (32%) in the standard group and 18 (15%) in the biomarker group have empirically recevied the antifungal treatment (difference 17%, 95% CI 4–26; *p* = 0.002).
Petitt et al. (2019) [48]	In an 811-bed acute care academic medical center, USA	Antimicrobial stewardship review of automated candidemia alerts using the epic stewardship module improves bundle-of-care adherence	2 years	No difference was observed in mortality	No difference was observed in the length of stay	No difference was observed in cost	Not reported	Not reported
Samura et al. (2020) [80]	Yokohoma general hospital, Japan	Before and after study pharmacist-led antifungal stewardship	8 years	Not reported	The days of therapy of antifungal drugs in the pre- and post-AFP groups was median 6.0 (interquartile range [IQR] 0.3–15.7) and median 3.4 (IQR 1.9–3.4) per 1000 patient-days, respectively; there was a significant decrease in the post-AFP group (*p* < 0.001).	The antifungal drugs expenditure as outcome parameter, in the pre and post AFP groups was (9390.5 ± 5687.1 and 5930.8 ± 4687.0 USD), respectively; there was a significant decrease in the post-AFP group (*p* = 0.002).	The cumulative optimal antifungal drug use rate markdly increased in the post-AFP group (*p* = 0.025)	Not reported
Hare et al. (2020) [60]	Tertiary referral centre, Ireland	Cohort study evaluating impact and safety of a multi-faceted diagnostic-driven antifungal stewardship on antimicrobial consumption	2 years	No change in mortality was reported	In compliant episodes without IC, median antifungal stewardship duration was 5.5 days [IQR 4–7]	Not reported	Not reported	Not reported
Machado et al. (2021) [63]	1250-bed tertiary care hospital, Spain	Before and after study, Utility of 1, 3 β-d-glucan assay in antifungal stewardship programs for oncologic patients	6 years	All cause mortality was similar in both periods (44.7% vs. 34.8%; *p* = 0.16), and no observable differences were found for IFI-related mortality (10.6% vs. 4.5%; *p* = 0.17)	Median days of treatment for empirical antifungal courses decreased from 9 (IQR 4–14) in the PRE-period to 5 (IQR 2–11) in the POST-period (*p* = 0.04)	Not reported	Not reported	The caspofungin use in the post period (21.2% vs. 6.2%; *p* = 0.002) was reduced, while fluconazole prescriptions was increased in the post period (18.8% vs. 45.5%; *p* < 0.001)
Stueber et al. (2020) [52]	971-bed community hospital, USA	Retrospective, observational study, Utilization and impact of a rapid Candida panel on antifungal stewardship program	3 years	Not reported	Fewer days of antifungal therapy	Not reported	Antifungal optimization occurred in 54% of patients who had antifungal orders	Not reported
Kawaguchi et al. (2019) [75]	Tertiary care hospital, Japan	Before and after study, The effects of antifungal stewardship programs	5 years	A reducing trend was apparent in patients with candidemia in the 30-day mortality (40.9% vs. 30.0%, *p* = 0.414) and in-hospital mortality (63.6% vs. 36.7%, *p* = 0.054)	Monthly average days of therapy per 1000 patient-days was markdly lower in the intervention group (15.1 ± 3.1 vs. 12.7 ± 4.3, *p* = 0.009)	The antifungals cost reduced over the 3 years period by $260,520 (13.5%).	Not reported	No significant difference was apparent in the defined daily doses per 1000 patient-days (23.3 ± 8.0 vs. 20.4 ± 10.8, *p* = 0.251) between the groups
Patch et al. (2018) [47]	A multi-hospital community health system, USA	A multi-hospital community health system on time to initiation of antifungal therapy in candidaemic patients as well as the utilization of micafungin	2 years	There were no statistically significant differences in all-cause 30 day readmissions or in mortality	There was no significant differences in length of hospital or ICU stay,	Not reported	There was a significant decrease in time to appropriate therapy in the post-T2Candida group (34 vs. 6 h, *p* = 0.0147). Empirical antifungal therapy was avoided in 58.4% of T2Candida-negative patients.	Not reported
Mendoza-Palomar et al. (2021) [67]	Tertiary care centre, Spain	describe the use and appropriateness of AFS in a high complexity paediatric centre	3 months	Not reported	Not reported	Not reported	The use of AFS without paediatric approval accounted for 8/24 inappropriate prescriptions.	Not reported
